# Supplementary material for: Multi‐dataset identification of innovative feature genes and molecular mechanisms in keratoconus
Source: J Cell Mol Med. 2024 Sep 19;28(18):e70079. doi: 10.1111/jcmm.70079 (PMC11412914; doi:10.1111/jcmm.70079)
Supplement: Supplementary file 2 — Material S2. [file JCMM-28-e70079-s002.zip › 7.GSEA analysis/3.DBNDD1/KEGG_RENAL_CELL_CARCINOMA.html]

Details for gene set KEGG\_RENAL\_CELL\_CARCINOMA[GSEA]

|  || Dataset | DBNDD1.group.cls#h\_versus\_l |
| Phenotype | group.cls#h\_versus\_l |
| Upregulated in class | l |
| GeneSet | KEGG\_RENAL\_CELL\_CARCINOMA |
| Enrichment Score (ES) | -0.36757255 |
| Normalized Enrichment Score (NES) | -1.1545874 |
| Nominal p-value | 0.34607646 |
| FDR q-value | 0.7035723 |
| FWER p-Value | 0.995 |
Table: GSEA Results Summary

  

Fig 1: Enrichment plot: KEGG\_RENAL\_CELL\_CARCINOMA      
 Profile of the Running ES Score & Positions of GeneSet Members on the Rank Ordered List

  

| SYMBOL | TITLE | RANK IN GENE LIST | RANK METRIC SCORE | RUNNING ES | CORE ENRICHMENT || 1 | PIK3R5 | na | 35 | 0.708 | 0.1090 | No |
| 2 | HGF | na | 3455 | 0.233 | 0.0466 | No |
| 3 | PIK3R1 | na | 6912 | 0.126 | -0.0335 | No |
| 4 | SOS1 | na | 8586 | 0.093 | -0.0673 | No |
| 5 | CREBBP | na | 9242 | 0.082 | -0.0734 | No |
| 6 | AKT2 | na | 9388 | 0.080 | -0.0652 | No |
| 7 | MAPK3 | na | 9865 | 0.073 | -0.0675 | No |
| 8 | MAP2K2 | na | 10931 | 0.059 | -0.0891 | No |
| 9 | PIK3R2 | na | 11699 | 0.050 | -0.1034 | No |
| 10 | RBX1 | na | 13500 | 0.034 | -0.1501 | No |
| 11 | SOS2 | na | 13580 | 0.033 | -0.1471 | No |
| 12 | TGFB3 | na | 13624 | 0.033 | -0.1433 | No |
| 13 | KRAS | na | 14127 | 0.029 | -0.1532 | No |
| 14 | PIK3R3 | na | 14144 | 0.029 | -0.1492 | No |
| 15 | PAK4 | na | 14233 | 0.028 | -0.1473 | No |
| 16 | RAC1 | na | 14759 | 0.025 | -0.1587 | No |
| 17 | PAK6 | na | 14984 | 0.023 | -0.1616 | No |
| 18 | BRAF | na | 15471 | 0.019 | -0.1726 | No |
| 19 | FH | na | 16255 | 0.014 | -0.1929 | No |
| 20 | VHL | na | 16864 | 0.010 | -0.2089 | No |
| 21 | RAP1A | na | 16884 | 0.010 | -0.2079 | No |
| 22 | PIK3CA | na | 17073 | 0.009 | -0.2119 | No |
| 23 | VEGFB | na | 17569 | 0.006 | -0.2253 | No |
| 24 | RAP1B | na | 18018 | 0.003 | -0.2378 | No |
| 25 | PIK3CB | na | 18338 | 0.000 | -0.2469 | No |
| 26 | HRAS | na | 18984 | -0.004 | -0.2649 | No |
| 27 | RAF1 | na | 19005 | -0.005 | -0.2647 | No |
| 28 | AKT1 | na | 19066 | -0.005 | -0.2657 | No |
| 29 | CUL2 | na | 19122 | -0.005 | -0.2664 | No |
| 30 | ARNT | na | 19123 | -0.005 | -0.2656 | No |
| 31 | TGFB2 | na | 19303 | -0.007 | -0.2697 | No |
| 32 | PTPN11 | na | 19596 | -0.009 | -0.2766 | No |
| 33 | EGLN2 | na | 21217 | -0.024 | -0.3196 | No |
| 34 | GRB2 | na | 22294 | -0.035 | -0.3452 | No |
| 35 | ARNT2 | na | 22414 | -0.036 | -0.3430 | No |
| 36 | CDC42 | na | 22626 | -0.039 | -0.3431 | No |
| 37 | EPAS1 | na | 22638 | -0.039 | -0.3374 | No |
| 38 | MAPK1 | na | 22705 | -0.040 | -0.3331 | No |
| 39 | TGFA | na | 22844 | -0.041 | -0.3307 | No |
| 40 | CRK | na | 23147 | -0.045 | -0.3324 | No |
| 41 | PAK3 | na | 23893 | -0.055 | -0.3454 | No |
| 42 | JUN | na | 24077 | -0.057 | -0.3417 | No |
| 43 | EP300 | na | 24292 | -0.060 | -0.3386 | No |
| 44 | PAK2 | na | 24360 | -0.061 | -0.3310 | No |
| 45 | ARAF | na | 24817 | -0.068 | -0.3335 | No |
| 46 | PIK3CD | na | 24971 | -0.070 | -0.3270 | No |
| 47 | EGLN1 | na | 25375 | -0.077 | -0.3268 | No |
| 48 | RAPGEF1 | na | 26791 | -0.103 | -0.3515 | Yes |
| 49 | SLC2A1 | na | 26795 | -0.103 | -0.3355 | Yes |
| 50 | CRKL | na | 27010 | -0.108 | -0.3249 | Yes |
| 51 | NRAS | na | 27014 | -0.108 | -0.3082 | Yes |
| 52 | PIK3CG | na | 27455 | -0.118 | -0.3026 | Yes |
| 53 | GAB1 | na | 27482 | -0.118 | -0.2849 | Yes |
| 54 | PAK1 | na | 28053 | -0.132 | -0.2808 | Yes |
| 55 | HIF1A | na | 28452 | -0.142 | -0.2702 | Yes |
| 56 | FLCN | na | 28478 | -0.143 | -0.2487 | Yes |
| 57 | MAP2K1 | na | 29246 | -0.164 | -0.2453 | Yes |
| 58 | PGF | na | 29370 | -0.168 | -0.2227 | Yes |
| 59 | MET | na | 29439 | -0.170 | -0.1982 | Yes |
| 60 | VEGFA | na | 30038 | -0.189 | -0.1860 | Yes |
| 61 | TGFB1 | na | 30914 | -0.218 | -0.1774 | Yes |
| 62 | VEGFC | na | 31739 | -0.251 | -0.1622 | Yes |
| 63 | EGLN3 | na | 33519 | -0.372 | -0.1557 | Yes |
| 64 | AKT3 | na | 33656 | -0.385 | -0.0998 | Yes |
| 65 | ETS1 | na | 33844 | -0.409 | -0.0416 | Yes |
| 66 | PDGFB | na | 34011 | -0.431 | 0.0207 | Yes |
Table: GSEA details [plain text format]

  

Fig 2: KEGG\_RENAL\_CELL\_CARCINOMA      
 Blue-Pink O' Gram in the Space of the Analyzed GeneSet

  

Fig 3: KEGG\_RENAL\_CELL\_CARCINOMA: Random ES distribution      
 Gene set null distribution of ES for **KEGG\_RENAL\_CELL\_CARCINOMA**
